# The Status of *Setaria viridis* Transformation: *Agrobacterium*-Mediated to Floral Dip

**DOI:** 10.3389/fpls.2018.00652

**Published:** 2018-05-25

**Authors:** Joyce Van Eck

**Affiliations:** ^1^Boyce Thompson Institute, Ithaca, NY, United States; ^2^Plant Breeding and Genetics Section, School of Integrative Plant Science, Cornell University, Ithaca, NY, United States

**Keywords:** AGL1, *Agrobacterium tumefaciens*, green bristlegrass, *in planta* transformation, mature-seed derived callus, monocot transformation, *Setaria italica*

## Abstract

*Setaria viridis* has many attributes, including small stature and simple growth requirements, that make it attractive as a model species for monocots. Genetic engineering (transformation) methodology is a key prerequisite for adoption of plant species as models. Various transformation approaches have been reported for *S. viridis* including tissue culture-based and *in planta* by *Agrobacterium tumefaciens* infection of floral organs referred to as the floral dip method. The tissue culture-based method utilizes *A. tumefaciens* infection of mature seed-derived callus with subsequent recovery of stable transgenic lines. Vectors found to be most effective contain the hygromycin phosphotransferase selectable marker gene driven by either *Panicum virgatum* or *Zea mays* ubiquitin promoters. As for the floral dip method, there are two reports based on *Agrobacterium* infection of young *S. viridis* inflorescences. Plants were allowed to mature, seeds were collected, and analysis of the progeny verified the presence of transgenes. Each transformation approach, tissue culture-based and floral dip, has advantages and disadvantages depending on the expertise of personnel and resources available. While the tissue culture-based method results in a higher transformation efficiency than floral dip, implementation requires a specific technical skillset that limits availability of experienced personnel to successfully perform transformations. Less technical experience is required for floral dip; however, a lack of high-quality growth chambers or greenhouses that provide the necessary optimum growing conditions would reduce an already low transformation efficiency or would not result in recovery of transgenic lines. An overview of transformation methods reported for *S. viridis* is presented in this review.

## Introduction

*Setaria viridis* is the weedy, wild ancestor of the domesticated *Setaria italica* (foxtail millet), which is an important food crop in some eastern Asian countries (Dekker, 2003). *S. viridis*, also known as green bristlegrass, green millet, and green foxtail, is a self-pollinating, small, diploid annual grass (Defelice, 2002). The short stature (10–15 cm) of *S. viridis* compared to the larger size of *S. italica* and other closely related Poaceae family members such as maize, *Miscanthus*, and sugarcane is an attribute that sparked interest in its development as a model species for important food and bioenergy crops (Brutnell et al., 2010). Being that *S. viridis* is a C4 photosynthesis grass, further interest in *S. viridis* as a model monocot species was driven by the potential of its usefulness in advancing knowledge of the cellular and biochemical mechanisms of C4 photosynthesis (Brutnell et al., 2015).

*Setaria viridis* has a small genome size (510 Mb) for which genome sequence is available, it has a short generation time of 6–9 weeks, and with its simple growth requirements, large populations can be grown under greenhouse and growth chamber conditions (Li and Brutnell, 2011; Bennetzen et al., 2012). Li and Brutnell (2011) showed that under a short-day photoperiod, *S. viridis* grows to less than 10 cm in height at flowering. *S. viridis* also has a high level of seed production with the potential to produce approximately 34,000 seeds per plant under suitable growing conditions (Stevens, 1932).

Development of genetic engineering methodology, often referred to as transformation, is a key factor when building a model species platform and this was certainly true for establishing *S. viridis* as a model. The ability to introduce or modify genes plays a critical role in gene identification and elucidation of function, which leads to an enhanced understanding of gene networks and mechanisms. Over the past three decades, various techniques have been developed to introduce genes of interest into plant cells. These gene transfer techniques include: direct DNA uptake into protoplasts (Paszkowski et al., 1984), delivery of DNA by particle bombardment (biolistics; Sanford et al., 1987), and by *Agrobacterium tumefaciens*-mediated methods (Gelvin, 2003). There are advantages and disadvantages of each of these approaches that need to be taken into consideration when choosing gene transfer methods for particular plant species. Direct DNA uptake into protoplasts requires methods for plant regeneration, which can be difficult depending on the plant species. For the biolistics method, a gene gun and related supplies are required, which can make this method cost prohibitive for many research groups. In addition, copy number of the introduced transgene needs to be taken into consideration from the standpoint of potential complications with transgene expression, namely, gene silencing (Tang et al., 2007). *Agrobacterium*-mediated transformation results in a lower transgene copy number than delivery by biolistics and the newly reported pollen magnetofection transformation (Zhao et al., 2017). As for *Agrobacterium*-mediated transformation, it is important that the plant material that works best for plant regeneration under tissue culture conditions is amenable to infection yet not adversely affected by the bacterial infection.

*Agrobacterium tumefaciens* is a soil-borne plant pathogenic bacterium that engineers infected cells to produce metabolites needed to support its growth. More than 30 years ago, researchers modified a strain of *A. tumefaciens* to investigate its utility for transformation of petunia, tobacco, and tomato (Horsch et al., 1985). In the intervening years, there has been a significant number of reports of genetic engineering of both dicots and monocots using *A. tumefaciens*; however, initially, monocots proved to be more problematic (Sood et al., 2011). This was partially due to low efficiency of plant regeneration at that time and, in addition, monocots are not natural hosts of *A. tumefaciens*. Over time, strategies were designed to overcome the barriers of plant regeneration and infection which led to more routine methods for *Agrobacterium*-mediated transformation of several monocot species. Various tissues including immature embryos (Ishida et al., 2007) and regenerable callus derived from immature embryos, immature inflorescences, and mature seeds (Lee et al., 2004; Burris et al., 2009; Song et al., 2011) have been used for *A. tumefaciens*-mediated transformation of monocots. Efforts by researchers worldwide led to advancement of *Agrobacterium* and biolistics approaches for monocot transformation that allowed genetic engineering of the major cereal crops (maize, rice, wheat, barley, sorghum, oats, and millets), which represents roughly two-thirds of the world food supply (Ji et al., 2013). For *S. viridis* transformation methods, there are reports of *Agrobacterium*-mediated transformation of mature seed-derived callus (Brutnell et al., 2010; Van Eck and Swartwood, 2015; Van Eck et al., 2017) and non-tissue culture methods (*in planta*) through infection of immature inflorescences followed by recovery of transgenic seed (Martins et al., 2015a; Saha and Blumwald, 2016).

The purpose of this review is to provide an overview of information presented at the Second International *Setaria* Genetics Conference on the status of genetic engineering approaches developed for *S. viridis* (Zhu et al., 2017). The first report of transformation was based on *Agrobacterium* infection of mature-seed derived callus of *S. viridis* A10.1 (Brutnell et al., 2010). Modifications of this method and strategies to improve the efficiency including decreasing the time for recovery of transgenic lines have been made. In addition to details regarding the tissue-culture-based approach for transformation, a summary of two reports on infection of floral organs in immature inflorescences, commonly referred to as the floral dip method, will also be described here. As evidenced from the collection of reports in this research topic, *S. viridis* has proven to be an effective model and the availability of engineering strategies has contributed to its utility to advance investigations.

## *Agrobacterium Tumefaciens*- Mediated Transformation of Mature Seed-Derived Callus

As indicated earlier, methods for *Agrobacterium*-mediated transformation of *S. viridis* were first reported in 2010 (Brutnell et al., 2010). In brief, the method was based on infection of mature seed-derived callus of *S. viridi*s A10.1 with the *A. tumefaciens* strain AGL1 that contained a vector optimized for monocot transformation. The time from infection of callus to recovery of mature T1 seed was approximately 4 months. Depending on the composition of the selectable marker gene cassette in the vectors used for transformation, the transformation efficiency, based on infection of the total number of mature seed-derived calli per experiment, ranged from 5 to 15% (Van Eck et al., 2017). Transformation efficiency is defined as the percentage of infected material that gives rise to at least one independent transgenic line.

The most effective vectors for *S. viridis* transformation were those that contained the hygromycin phosphotransferase selectable marker gene (*hpt*), which confers resistance to the antibiotic hygromycin. The promoter driving *hpt* was also found to be key for efficient transformation. Vectors that contained *hpt* driven by a monocot promoter resulted in recovery of a greater number of transgenic lines compared to vectors where *hpt* expression was under the control of promoters best suited for dicots (Van Eck et al., 2017). In addition, those vectors that had an intron in the *hpt* gene resulted in a greater number of transgenic lines than when an intron was not present in the gene. Similar findings were also reported when a vector with an intron-containing *hpt* was used for rice transformation (Wang et al., 1998). Gene constructs designed with the following vectors were found to be best for *S. viridis* transformation: pWBVec8 (Wang et al., 1998), pOL001 (Vogel and Hill, 2008), pMDC (Curtis and Grossniklaus, 2003), and pANIC (Mann et al., 2012).

In addition to the *hpt* selectable marker gene, vectors used for the development of the *S. viridis* transformation methodology also contained the beta-glucuronidase (GUS) and green fluorescent protein (GFP) reporter genes (Martins et al., 2015b; Van Eck and Swartwood, 2015).

Additional modifications of the *Agrobacterium*-mediated transformation methodology of *S. viridis* A10.1 were also reported by a second group (Martins et al., 2015b). Martins et al. (2015b) followed similar methodology as reported by Van Eck et al. (2017). A comparison of the primary components that make up the various reported transformation methods (Martins et al., 2015b; Van Eck and Swartwood, 2015; Van Eck et al., 2017) is outlined in **Table 1**. The vectors used for transformation, more specifically the type of selectable marker gene and its promoter contained in the vector, were the key parameters that influenced transformation efficiency in the methods described by Martins et al. (2015b). The vector designated p7U resulted in the lowest transformation efficiency at 6%. p7U contained the bialaphos resistance selectable marker gene (*bar*) under control of the ubiquitin promoter from maize. The vectors used for their transformation efforts that resulted in higher transformation efficiencies than p7U contained *hpt* under control of either the CaMV 35S promoter or an enhanced version. In addition, *hpt* in the vectors that resulted in higher levels of transgenic line recovery was either codon optimized for expression in monocots or contained an intron. Wang et al. (1998) demonstrated that an intron-containing version of *hpt* resulted in higher transformation efficiencies for rice than a non-intron-containing version. This result combined with those reported by Van Eck et al. (2017) points to the importance of vector selection, namely, the composition of the selectable marker gene cassette, for *S. viridis* transformation. Researchers should be aware that perhaps a vector that works efficiently for transformation of one monocot species might not work efficiently in a different monocot.

**Table 1:** Comparison of parameters reported for *Agrobacterium tumefaciens*-mediated transformation of mature seed-derived callus of *Setaria viridis*.

Parameters	Van Eck et al.^1^	Martins et al.^1^
*Setaria viridis* accession	A10.1	A10.1
*Agrobacterium tumefaciens* strains	AGL1	EHA105
Medium components^1^	MS^2^ salts-based CIM^3^, zinc sulfate, maltose, and Gelzan	MS salts-based CIM, biotin, sucrose, and Phytagel
Age of callus infected	6–8-weeks-old	4–6-weeks-old
Selectable marker genes^4^	*hpt*	*hpt* and *bar*
Transformation efficiency^5^	A10.1: 5–15%	A10.1: 8–29%

*^1^For additional details on media components, see the following: Martins et al., 2015b; Van Eck and Swartwood, 2015; Van Eck et al., 2017. ^2^Murashige and Skoog salts’ formulation (Murashige and Skoog, 1962). ^3^CIM – callus induction medium. ^4^hpt – hygromycin phosphotransferase gene which confers resistance to hygromycin; bar – bialaphos resistance gene. ^5^Transformation efficiency: percentage of infected callus that gives rise to at least one independent transgenic line. The range in Setaria viridis transformation efficiency resulted from the composition of plant selectable marker gene cassette (gene and its promoter) present in the binary vectors used for transformation experiments.*

In the years since the first report by Brutnell et al. (2010) on *S. viridis*’ potential as a model that also included preliminary information on *Agrobacterium*-mediated transformation of mature seed-derived callus, some key factors related to callus generation and plant regeneration were identified that improved the efficiency of transgenic line recovery (Van Eck and Swartwood, 2015; Van Eck et al., 2017). One of these factors included the gelling agent used in the callus induction medium (CIM). When agar was used as the gelling agent in the CIM, the callus quality was poor and resulted in a low level of plant regeneration. However, the substitution of a gellan gum-type gelling agent (Phytagel or Gelzan) in the CIM greatly improved the callus quality and subsequent plant regeneration. Additional changes such as the substitution of maltose for sucrose, addition of ZnSO_4_ in the CIM and inclusion of an agar (Phytoblend) as the gelling agent in the plant regeneration medium instead of Phytagel or Gelzan enhanced the recovery of transgenic lines (Van Eck et al., 2017).

## Floral Dip Transformation

The ability to deliver genes of interest into plant cells through bypassing tissue culture-based methods, often referred to as *in planta* transformation, has advantages such as specialized equipment (e.g., laminar flow hood) is not required and the introduction of somaclonal variation, that can result in off-type plants, is avoided (Bairu et al., 2011). A non-tissue culture-based method that involves application of *A. tumefaciens* containing gene constructs of interest to floral organs is referred to as floral dip transformation. This method is routinely used for Arabidopsis transformation. Development and modifications of this approach greatly advanced the utilization of Arabidopsis as a model (Clough and Bent, 1998; Somerville and Koornneef, 2002; Zhang et al., 2006). Floral dip methods have also been reported for other plant species including wheat (Agarwal et al., 2009), maize (Mu et al., 2012), and rice (Ratanasut et al., 2017). However, the floral dip transformation approach for these species has not been widely adopted and they are generally transformed by *Agrobacterium*-mediated tissue-culture-based methods or biolistics.

There are two reports for floral dip transformation of *S. viridis* (Martins et al., 2015a; Saha and Blumwald, 2016). In brief, both methods involved exposure of immature inflorescences of *S. viridis* to *A. tumefaciens* containing a gene construct followed by recovery of mature seed that gave rise to plants that were confirmed to be transgenic. Martins et al. (2015a) indicated that 1.5–2 months following floral dipping were required to identify T1 seeds by assessment for red fluorescence. Saha and Blumwald (2016) reported recovery of fertile, transgenic plants within 8–10 weeks of floral dip. Various parameters were evaluated to develop the best approach for this *in planta* transformation method in *S. viridis* and a comparison of parameters reported by both groups is presented in **Table 2**. Martins et al. (2015a) focused their efforts on *S. viridis* A10.1, whereas Saha and Blumwald (2016) utilized three different genotypes: A10.1, 132, and 98HT-80. The 98HT-80 genotype resulted in the highest transformation efficiency at 0.8% and the efficiency of A10.1 reported by both groups was approximately 0.6%. Approaches for determining transformation efficiency differed in the reports. Martins et al. (2015a) based their transformation efficiency on the number of PCR-verified transgenic plants recovered from germination of 1000 seeds harvested from *Agrobacterium*-infected immature inflorescences. However, Saha and Blumwald (2016) based the transformation efficiency on the number of hygromycin-resistant plants recovered following germination of a pre-determined number of seeds on hygromycin-containing medium. These *in planta* methods provide alternative approaches to the tissue culture-based *Agrobacterium* method of infection of callus derived from mature seeds of *S. viridis*.

**Table 2:** Comparison of parameters reported for floral dip transformation of *Setaria viridis*.

Parameters	Martins et al., 2015b	Saha and Blumwald, 2016
*Setaria viridis* accession	A10.1	A10.1, 132, 98HT-80
Inflorescence stage	Boot stage	5-days-old spikes
*Agrobacterium tumefaciens* strain	AGL1	AGL1, EHA105, GV3101, and LBA4404
Vectors	pANIC 6A	Multiple
Selectable marker gene	*hpt*^1^	*hpt*
Transformation efficiency	0.6%^2^	0.5–0.8%^3^

*^1^hpt – hygromycin phosphotransferase gene which confers resistance to hygromycin. ^2^Transformation efficiency based on the number of PCR-verified transformants/1000 mature seeds collected from infiltrated immature inflorescences. ^3^Transformation efficiency based on the number of hygromycin-resistant seedlings recovered after germination on hygromycin-containing medium/total number of seeds tested. The range of transformation efficiency is a result of the effects of different parameters tested.*

## Conclusion

Robust methods for genetic engineering whether it be tissue culture-based or *in planta* are critical for dissecting the intricacies of gene function and networks. Knowledge gained from gene function studies helps guide development of strategies for crop improvement. This is true whether these gene function studies are performed directly in crop species or models that result in translatable information to crops. The genetic engineering resources described here for *S. viridis* combined with attributes that make it an ideal model system have advanced many studies that might not otherwise have been realized (Huang et al., 2016). The availability of tissue culture-based and *in planta* transformation methods provides options for performing gene function studies in *S. viridis* and allows application of new technologies such as gene editing. While there are advantages and disadvantages as described in this review for each transformation method, researchers need to investigate the different methods to determine the approach that is most feasible and reproducible in their labs. Advances in genetic engineering methods, such as the recently reported pollen magnetofection in cotton (Zhao et al., 2017), along with continued development of genetic and genomic resources will greatly benefit the *Setaria* research community.

## Author Contributions

The author confirms being the sole contributor of this work and approved it for publication.

## Conflict of Interest Statement

The author declares that the research was conducted in the absence of any commercial or financial relationships that could be construed as a potential conflict of interest.
